# Draft Genome Sequence of the Sulfonamide Antibiotic-Degrading *Microbacterium* sp. Strain C448

**DOI:** 10.1128/genomeA.01113-13

**Published:** 2014-02-13

**Authors:** Fabrice Martin-Laurent, Romain Marti, Nicholas Waglechner, Gerard D. Wright, Edward Topp

**Affiliations:** a INRA, UMR 1347 Agroecologie, Dijon, France; b Agriculture and Agri-Food Canada, London, ON, Canada; c MG DeGroote Institute for Infectious Disease Research, McMaster University, Hamilton, ON, Canada

## Abstract

We report the draft genome sequence of *Microbacterium* sp. strain C448, isolated from agricultural soil with a decade of exposure to veterinary antibiotics on the basis of using sulfamethazine and other antibiotics as the sole sources of carbon. The genome sequence revealed that strain C448 harbors several antibiotic resistance genes, including *sulI*.

## GENOME ANNOUNCEMENT

Antibiotics used in commercial agriculture can reach land through the application of animal manures. In order to assess whether exposure to environmentally relevant antibiotic concentrations promoted antibiotic resistance or had other impacts on soil microbial communities, a long-term field experiment was initiated in 1999 in London, Ontario, Canada. Plots cropped to soybeans have since received an annual spring application of a mixture of sulfamethazine, chlortetracycline, and tylosin, commonly used in swine production. Importantly, exposure to antibiotics resulted in a marked reduction in the persistence of sulfamethazine and tylosin (1). Strain C448, identified as a *Microbacterium* sp. on the basis of 16S rRNA gene sequence analysis (Genbank accession number JQ804844), was isolated from plots treated with 10 mg/kg antibiotics on the basis of the ability to metabolize sulfamethazine (1). Strain C448 mineralizes the phenyl ring of sulfonamide antibiotics to CO_2_ and excretes the rest of the molecule as a dead-end product. The 16S rRNA gene sequence of strain C448 shares 99% identity with that of *Microbacterium* sp. strain BR1, isolated from an acclimated wastewater bioreactor and able to mineralize sulfamethoxazole (2, 3). Strain C448 shares 99% identity with *Microbacterium lacus* strain SDZm4, a sulfadiazine-degrading bacterium isolated from a sample of German soil with a history of manure fertilization from sulfadiazine-medicated pigs (4). Overall, the recent isolation of sulfonamide-degrading *Microbacterium* isolates from disparate environments suggests their widespread importance in environmental dissipation of these drugs.

Here, the whole genome of strain C448 was sequenced by paired-end (PE) Illumina sequencing (Illumina, Inc.). Sequencing yielded 592,448 pairs of 150-bp high-quality filtered reads, totaling 177,734,400 bp, providing approximately 53-fold genome coverage. Using the Velvet 1.2.06 program with automatic expected coverage and coverage cutoff parameters and the setting *k* = 31, we assembled the reads into a consensus sequence of 3.29 Mbp (split into 268 contigs). The *N*_50_ is 31,621 bp for this assembly, meaning that half the reported genome (3.29 Mbp) occurs on contigs of 31,621 bp or larger, and the largest contig is 104,475 bp. Annotation of the C448 draft genomic sequence was performed using the BioProject facilities at the GenBank database (under the identifier B234, accession numbers PRJNA170195 and ID170195) and the Microbial Genome Annotation and Analysis Platform (MicroScope platform [http://www.genoscope.cns.fr/agc/microscope/]) of the Genoscope (the French National Sequencing Center) (5). The draft genome sequence of strain C448 consists of 3,273,510 bases, with a GC content of 67.39%. The analysis of the C448 genome revealed that it contains numerous resistance genes, including *sulI*, encoding dihydropteroate synthase type 1. The *int1* and *parA* genes, coding for an integrase and a resolvase, are located close to *sul1*, forming an island of resistance to sulfamethazine.

### Nucleotide sequence accession numbers.

The draft sequence of *Microbacterium* sp. strain C448 has been deposited in the European Nucleotide Archive under the accession numbers HG779581 through HG779847 and CBVQ010000001 through CBVQ010000268. This paper is based on the analysis of the first version of this draft genome sequence.
